# Extraosseous Ewing Sarcoma in a Pelvic Region: A Case Report

**DOI:** 10.31729/jnma.7523

**Published:** 2022-07-31

**Authors:** Sushma Gurung, Sagun Thapa, Shristi Gautam

**Affiliations:** 1 Department of Paediatric Haematology and Oncology, Bhaktapur Cancer Hospital, Dudhpati, Bhaktapur, Nepal

**Keywords:** *Ewing sarcoma*, *neoadjuvant chemotherapy*, *radiation therapy*

## Abstract

Ewing sarcoma is the second most common malignant bone tumour in children. It rarely originates from extraskeletal soft tissue sites such as the upper thigh, buttocks, upper arm and shoulder. Primary extraosseous Ewing sarcoma located in the pelvic region is rare. We report a 17-year-female who had gradual onset of progressive lower abdominal mass and pain. A computed tomography scan revealed well defined lobulated heterogeneously enhancing lesion noted in the pelvic region measuring approximately 12.9 x 9.8 x 9.3 cm. Incisional biopsy showed a small round blue cell tumour which was strongly positive for Cluster of Differentiation 99, vimentin, Friend Leukaemia Integration 1 with 40% Ki-67. Following treatment with chemotherapy, surgery and radiotherapy, there was complete resolution of the tumour. Although extraosseous Ewing sarcoma is rare, it can occur virtually in any soft tissue site. Therefore, clinicians need to distinguish it from soft tissue sarcoma because rapid progression, early diagnosis and timely treatment are crucial for a favourable prognosis.

## INTRODUCTION

Ewing Sarcoma (ES) is the second most common primary bone malignancy in the paediatric population. It is characterized by poorly differentiated, aggressive clinical features with a high rate of local recurrences and distant metastasis. Extraosseous Ewing sarcoma (EES) is an uncommon primary tumour of the soft tissues which accounts for 10-20% of cases of Ewing sarcoma Family of Tumour (EFT).^1^ The most common sites of EES include extremities, chest wall, paravertebral space and retroperitoneum but rare anatomic locations have been reported such as jejunum, kidneys, heart, vulva and larynx.^2,3^ EES in the pelvic region is also rare and here we represent a rare case of it.

## CASE REPORT

**Figure 1:**
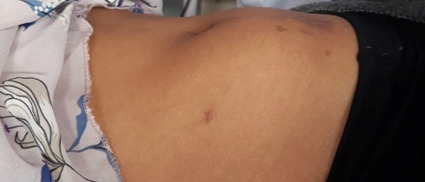
An ill-defined hard mass measuring approximately 10 x 10 cm.

A 17 years old female presented to a local hospital with a gradually progressive lump and pain in the lower abdomen for 4 weeks. On abdominal examination there was an ill-defined hard mass measuring approximately 10 x 10 cm palpable in the lower abdomen (Figure 1).

**Figure 2:**
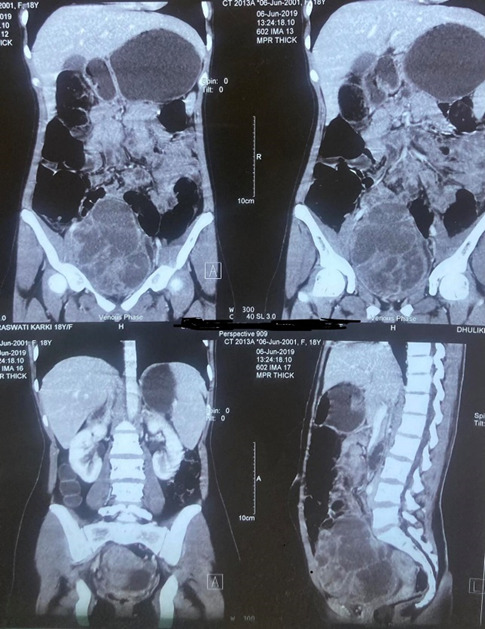
CECT scan at the time of diagnosis showed a large well defined lobulated heterogeneously enhancing lesion noted in the pelvic region with internal septation and solid components measuring approximately 12.9 x 9.8 x 9.3 cm.

Contrast-enhanced computed tomography (CECT) scan revealed a large well defined lobulated heterogeneously enhancing lesion noted in the pelvic region with internal septation and solid components measuring approximately 2.9 x 9.8 x 9.3 cm. The mass displaces the uterus posteriorly, urinary bladder is left posterolaterally and bowel loops superiorly. The lesion abuts the lower abdominal wall with minimal adjacent fat stranding noted (Figure 2).

Following an initial evaluation, the patient underwent an incisional biopsy which showed a large vascularised mass present in the pelvic region attached to the anterior abdominal wall and the pathological findings microscopically revealed a small round blue cell tumour with the differential diagnosis of desmoplastic small round cell tumour and extraskeletal Ewing sarcoma / primitive neuro-ectodermal tumors (PNET). Immunohistochemical stain identified tumour cells positive for Cluster of Differentiation 99 (CD99), vimentin, and Friend Leukaemia Integration 1 transcription factor (FLI-1) with 40% Ki-67 confirming the diagnosis of Ewing sarcoma. She was then referred to our centre for further management. Metastatic workup with a bone scan, bone marrow examination and chest CT scan was done which were normal. Therefore, the patient was diagnosed with localized extraosseous Ewing sarcoma. Neoadjuvant chemotherapy was initiated as per the Children's Oncology Group (COG) protocol which involved five drugs namely vincristine (V), doxorubicin (D) , cyclophosphamide (C), ifosfamide (I) and etoposide (E) (VDC alternating IE) every two weekly (dose-dense therapies).

**Figure 3:**
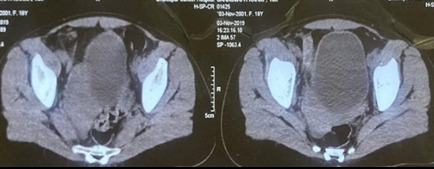
MRI scan after six cycles of NACT showed a well-defined heterogeneous density lesion with calcifications seen in the right peri vesical region, measuring approximately 5 x 1.7 cm.

Then, re-evaluation was done after six cycles of neoadjuvant chemotherapy (NACT). Magnetic resonance imaging (MRI) scan showed a significant reduction in the size of the lesion and a well-defined heterogeneous density lesion with calcifications was seen in the right peri vesical region measuring approximately 5 x 1.7 cm. It was abutting the urinary bladder medially and pelvic wall laterally. The mild fat stranding was seen adjacent to it (Figure 3).

Then, she was referred to surgical oncology department for local control (delayed surgery). She underwent surgery and excision of residual mass followed by 5500 cGy (180 cGy per fraction) of radiation therapy. Histopathological examination of the residual mass showed 50% viable tumour and scar tissue which was negative for malignancy. During the chemo-radiotherapy period, the patient was under closed follow-up with lab and radiographic imaging. Furthermore, chest and abdominopelvic CT scans and bone scans were negative for probable metastasis or involvement of any other sites. The last MRI of the pelvis (2 years after the completion of treatment) was done that dated no focal lesion in the pelvis and retroperitoneum to suggest recurrence.

## DISCUSSION

EES is far less frequent than skeletal Ewing sarcoma and may develop in soft tissues at any anatomical location, but the main sites of involvement are the trunk, paravertebral region and the extremities.^2^ EES in the pelvic region is rare, and only a few relevant cases have been reported in the previous literature. The most common clinical manifestation of EES is a rapidly growing mass with or without local pain and also, the radiologic characteristics of it are non-specific.^4^

Currently, diagnosis of EES is largely depends on pathological and immunohistochemical findings. Microscopically, EES represents distinctly monomorphous round blue cells, which were characterised by round oval sheet cells with primitive nuclei and clear cytoplasm. On an immunohistochemical stain, CD99 (a 32 KD cell surface glycoprotein encoded by the MIC2 gene) and FLI-1 represent the major diagnostic markers.^5^ FLI-1 is more specific for Ewing Sarcoma than CD99 but FLI-1 can be found also in lymphoblastic leukaemia, lymphomas and several soft-tissue sarcomas.^6^ More than 85% of patients with ewing sarcoma harbour the reciprocal translocation between the EWS and FLI-1 genes, t(11,22) (q24,q12), resulting in the EWS-FLI-1 fusion transcript.^7^ Molecular pathology by fluorescence in situ hybridisation (FISH) analysis or RT-PCR plays a crucial role in diagnosing Ewing sarcoma, particularly when microscopy and immunohistochemistry are not specific. In the present case, immunohistochemical stain showed strong positivity for CD99, vimentin, and FLI-1. As a result, the diagnosis of Ewing sarcoma was confirmed.

Tumours of the pelvis have a poorer prognosis when compared to other sites. Whether this is related to the challenge of achieving local control or the proximity to critical deep structures should be elucidated.^8,9^ The surgical approach should be combined with chemotherapy and additional radiotherapy in cases involving a marginal resection and/or poor histological response. Considering a few poor prognostic factors in our patient which include an older patient, a pelvic site with difficult margin delineation, the initial size of tumour more than 8 cm and poor response to chemotherapy, radiation therapy was added.

Notably, patients with EES tend to have a higher incidence of distant metastasis than skeletal ES patients it has been reported that 30-40% of patients had distant metastasis disease at the time of diagnosis.^10^ In our case, the patient did not have metastatic lesions and was treated successfully with chemotherapy, surgery, and radiation therapy. The patient remained free of disease through 2 years of follow-up until now after the completion of treatment.

EES is a rare subtype of the ewing sarcoma family of tumours and the pelvic site is the rarest. Due to the lack of specific clinical manifestation and radiological features, the definite diagnosis of EES is mainly based on pathological and immunohistochemical findings. Clinicians should recognize this entity from other soft tissue sarcomas. In general, the treatment approach of EES is also multimodality with chemotherapy, surgery and radiotherapy if needed. EES of the pelvic site has poorer prognosis when compared to other sites in view of challenges in achieving local control.
